# Draft Genome Sequence of *Rickettsia aeschlimannii*, Associated with *Hyalomma marginatum* Ticks

**DOI:** 10.1128/genomeA.00666-14

**Published:** 2014-07-24

**Authors:** Erwin Sentausa, Khalid El Karkouri, Caroline Michelle, Didier Raoult, Pierre-Edouard Fournier

**Affiliations:** Institut Hospitalo Universitaire Mediterranee-Infection, Aix-Marseille Université, URMITE, UM63, CNRS 7278, IRD 198, INSERM 1095, Marseille, France

## Abstract

*Rickettsia aeschlimannii* is a tick-associated human pathogen. We report here the draft genome of *R. aeschlimannii* strain MC16, isolated from *Hyalomma marginatum marginatum* ticks collected in Morocco.

## GENOME ANNOUNCEMENT

*Rickettsia* spp. are strictly intracellular bacteria that are associated with arthropods. Some of them cause human infections that are currently considered emerging and have been identified worldwide (1). *Rickettsia aeschlimannii* is a member of spotted fever group rickettsiae that was first isolated from *Hyalomma marginatum marginatum* ticks collected from camels in Morocco in 1992 (2). The first human infection was reported in 2002 in a French patient who had traveled to Morocco (3). *R. aeschlimannii* has since been reported to cause infections in South Africa (4), Tunisia (5), Algeria (6), and Greece (7). Here, we briefly describe the draft genome of *R. aeschlimannii* strain MC16^T^.

The MiSeq sequencer (Illumina, San Diego, CA) was used to sequence the genomic DNA of *R. aeschlimannii* MC16 (deposited in the Collection de Souches de l’Unite des Rickettsies under reference CSUR R8) using a mate-pair library. Quality trimming and *de novo* assembly of the reads were performed using the CLC Genomics Workbench version 6.0.1 (CLC bio, Aarhus, Denmark). The resulting contigs were reordered in Mauve version 2.3.1 (8) using *Rickettsia montanensis* strain OSU 85-930 as a reference genome (accession no. CP003340.1). Potential coding sequences (CDSs) were predicted using AMIGene (9), and the assignment of protein functions was performed by searching against the RickBase (10), GenBank, and Pfam (11) databases using BLASTp (12), while rRNAs, tRNAs, and other RNAs were identified using BLASTn, tRNAscan-SE version 1.21 (13), and RNAmmer 1.2 (14), respectively. Orthologous genes between *R. aeschlimannii* and *R. montanensis* were identified using OrthoMCL (15), with a BLASTp *E* value cutoff of 1 × 10^−5^ and the default Markov cluster algorithm (MCL) inflation parameter of 1.5.

The draft genome of *R. aeschlimannii* MC16 consists of 16 contigs ranging in size from 314 to 238,303 bases, resulting in a total genome of 1,312,196 nucleotides, with an average genome coverage of 145-fold and a G+C content of 32.2%. Two plasmids of 14,948 bp and 26,886 bp, respectively, were identified and exhibited identity matches of 96% (97% coverage; *E* value, 0.0) to *Rickettsia rhipicephali* strain 3-7-female6-CWPP plasmid pMCC_1 (accession no. CP003343.1) and 96% (27% coverage; *E* value, 0.0) to *Rickettsia monacensis* strain IrR/Munich plasmid pRM (accession no. EF564599.1), respectively. The chromosome contains 1,790 CDSs and, like other rickettsial genomes, 3 noncontiguous rRNAs (5S, 16S, and 23S rRNAs), 33 tRNAs, and 3 other RNAs. In addition, the two plasmids contain 23 and 44 CDSs but no RNAs.

The *R. aeschlimannii* chromosome exhibits a high level of synteny to *R. montanensis* but differs from it by three inversions of 66,949 bp, 3,404 bp, and 53,495 bp and the absence of the genes encoding the integration host factor beta-subunit (*himD*), large extracellular α-helical protein, 3-hydroxyacyl-coenzyme A (CoA) dehydrogenase (*fadB*), and competence protein F (*comF1* and *comF2*).

### Nucleotide sequence accession numbers.

This whole-genome shotgun project has been deposited at DDBJ/EMBL/GenBank under accession no. CCEA01000001 to CCEA01000014 (BioProject PRJEB6087), and the two plasmid sequences have been deposited under accession no. CCEB01000001 and CCEB01000002.
